# Alteration of Rat Fetal Cerebral Cortex Development after Prenatal Exposure to Polychlorinated Biphenyls

**DOI:** 10.1371/journal.pone.0091903

**Published:** 2014-03-18

**Authors:** Elise Naveau, Anneline Pinson, Arlette Gérard, Laurent Nguyen, Corinne Charlier, Jean-Pierre Thomé, R. Thomas Zoeller, Jean-Pierre Bourguignon, Anne-Simone Parent

**Affiliations:** 1 Developmental Neuroendocrinology unit, GIGA Neurosciences, University of Liège, CHU, Liège, Belgium; 2 Developmental Neurobiology unit, GIGA Neurosciences, University of Liège, CHU, Liège, Belgium; 3 Laboratory of Clinical, Forensic and Environmental Toxicology, University of Liège, CHU, Liège, Belgium; 4 Laboratory of Animal Ecology and Ecotoxicology (LEAE, CART), University of Liège, Liège, Belgium; 5 Biology Department, University of Massachusetts, Morrill Science Center, Amherst, Massachusetts, United States of America; Robert Debre Hospital, France

## Abstract

Polychlorinated biphenyls (PCBs) are environmental contaminants that persist in environment and human tissues. Perinatal exposure to these endocrine disruptors causes cognitive deficits and learning disabilities in children. These effects may involve their ability to interfere with thyroid hormone (TH) action. We tested the hypothesis that developmental exposure to PCBs can concomitantly alter TH levels and TH-regulated events during cerebral cortex development: progenitor proliferation, cell cycle exit and neuron migration. Pregnant rats exposed to the commercial PCB mixture Aroclor 1254 ended gestation with reduced total and free serum thyroxine levels. Exposure to Aroclor 1254 increased cell cycle exit of the neuronal progenitors and delayed radial neuronal migration in the fetal cortex. Progenitor cell proliferation, cell death and differentiation rate were not altered by prenatal exposure to PCBs. Given that PCBs remain ubiquitous, though diminishing, contaminants in human systems, it is important that we further understand their deleterious effects in the brain.

## Introduction

Polychlorinated biphenyls (PCBs) form a group of widespread environmental contaminants composed of 209 different congeners used in a wide variety of applications. Their production was banned in the 1970's but PCBs are still present in the environment due to their high stability [1]. Because they are strongly lipophilic, they accumulate in human and animal fat tissues and maternal milk [2], [3]. During the past decade, PCB concentrations were found to vary from 0.27 to 1.42 µg/L in human serum depending on the congener [3] and from 35.5 to 186.3 ppb (parts per billion) in maternal milk (reviewed in [2]). During fetal and early postnatal life, developing organisms are exposed to PCBs through placental and breast milk transfer [4]. Thus, the fetus and neonate are exposed to PCBs at a time of critical developmental importance.

PCB exposure, specifically during fetal development in humans, is associated with decreased intellectual quotient scores, impaired learning and memory, psychomotor difficulties and attention deficits [2], [5], [6]. Animal studies report similar findings [7], [8]. PCB exposure interferes with experience-dependent dendritic plasticity [9], impairs white matter development [10], [11] and increases cerebellar ryanodine receptor expression [12] in rodents, all of which may underlie the neurotoxic effects of PCBs on the developing brain.

We aimed at studying the effects of PCBs on the development of the cerebral cortex. The development of the cerebral cortex is a very complex process that follows tightly linked sequences of proliferation, cell cycle exit, cell migration to specific cell layers and neuronal differentiation. Progenitor cells proliferate in the ventricular and subventricular zones (VZ and SVZ, respectively). After symmetrical division to increase the progenitor pool of the VZ, cells begin to divide asymmetrically and produce two types of cells: radial glia cells and neuronal precursors. Some neural precursors exit the cell cycle to become projection neurons. Newborn projection neurons migrate along radial glia fibers to reach the cortical plate which is made of six cortical layers established by an inside-out migration, the deeper layers appearing before the superficial layers.

A dominant theory concerning the mechanism by which PCBs can influence brain development is that it interferes with TH action [13], [14] since PCB exposure causes a significant decrease in serum TH [15] and inhibits TH-dependent extension of Purkinje cell dendrites *in vitro* [16]. TH are known to regulate the cell cycle [17] as well as neuronal migration [18], neurite extension [19] and synaptogenesis [20], though these observations were not all made in the developing cortex. Therefore, we hypothesized that fetal exposure to PCBs could disrupt some aspects of cerebral cortical development through perturbation of one or several of these key processes. In this study we aimed at testing whether fetal exposure to Aroclor 1254, a commercial mixture of PCBs congeners similar to what is found in human maternal milk content, could interfere with development of the fetal rat cortex *in vivo* by focusing on three parameters potentially targeted by PCBs: neuronal progenitor proliferation, cell cycle exit and radial migration. In addition we investigated the serum level of thyroxine to approach the role played by TH in PCBs actions.

## Methods

### Ethics statement

All animal procedures were approved by the Ethical Committee at the University of Liège (Permit Number: 715).

### Animals and exposure

Timed pregnant wistar rats were housed individually under regulated temperature (22°C) and a 12-hr light/dark cycle. Pregnant rats were exposed orally to the commercial PCB mixture, Aroclor 1254 (Lot no. 124–191; AccuStandard, Inc., New Haven, CT), diluted in corn oil. Aroclor 1254 exhibit a congener profile similar to that found in human tissues and breast milk [21]. Dams were exposed daily (2:00–3:00 PM) to either Aroclor 1254 (6 mg/kg/day) or vehicle (corn oil), injected in one third of a wafer (Delacre, Groot-Bijgaarden, Belgium), from the sixth day of gestation (E6) until the last day of gestation. The doses were adjusted every other day to account for changes in body weight of the dams. Dams were anaesthetized with isoflurane (Isoflo, Abbott, UK) and trunk blood was collected after decapitation. Dams were divided into groups depending on age at sacrifice and exposition. Dams were sacrificed either at E17 or E19 to study cell proliferation, S phase and cell cycle exit, at E17 for cell death and neuronal differentiation rate and at E20 for neuronal migration, laminar organization and radial glia study. For each experiment, 3–6 embryos per group were used. Each embryo came from a different dam in order to avoid litter effects.

### PCB levels in dam serum and fetal brains

In order to appreciate Aroclor 1254 exposure of the dams and embryos, five individual PCB congeners (PCB 101, 118, 138, 153 and 180) that are the most abundant components of Aroclor 1254 (A1254) were quantified in the serum of control and exposed pregnant rats at E19. A chromatography in gaseous phase coupled to mass spectrometry was used to measure serum PCBs levels (toxicology laboratory, CHU, University of Liège) [22]. In addition, concentration of those congeners was measured in the whole fetal brain from Aroclor 1254-exposed and control fetuses obtained at E19. PCB congeners were analyzed in fetal brains after extraction of lipids with hexane using an accelerated solvent extractor (ASE) (Dionex 200, Sunnyvale, USA). Solvent was evaporated using a Turbovap LV (Zymarck, Hopkinton, MA, USA) until a constant weight was obtained. PCBs were extracted according to a method previously described by Debier et al. [23]. PCBs were finally separated by progressive temperature increase, identified according to their retention time and quantified thanks to the software Chrom-Card for Windows as described in Schnitzler et al. [24]

### T4 serum concentrations

Total and free serum T4 concentrations were measured in pregnant rat on the 20^th^ day of gestation using a radioimmunoassay kit (total T4: MP biomedical 06B254011, Solon, OH, USA, free T4: beckman coulter IM1363 Brea, CA, USA). Total and free T4 were measured in duplicate in 25 µl of rat serum; standards were run in triplicates. The detection limit, which was the value assigned to the undetectable samples, was 20 ng/ml for total T4 and 2.3 pg/ml for free T4.

### BrdU administration

The thymidine analogue 5-bromo-2′-deoxyuridine (BrdU) labels cells in S-phase of the cell cycle at the time of injection. BrdU (Sigma, St. Louis, MO) was administered intraperitoneally (50 mg/kg in one single injection) to the dams exposed to corn oil or Aroclor 1254. BrdU stock was prepared in 0.9% saline solution with NaOH 7 mM. In order to study cell proliferation in the VZ/SVZ of the fetal cortex, BrdU injections to the dams were done 1 h before sacrifice at E17 or E19. In order to study cell cycle exit in the fetal cortex, BrdU injections to the dams were done on E16 or E18, 24 h before sacrifice. Since Ki67 is a cellular marker labeling all proliferating cells, the combination of a staining for BrdU and Ki67 allowed a relative quantification of cortical progenitors having left the cell cycle (Ki67 negative cells) 24 h after being marked by BrdU [25]. Pregnant females were injected with a single dose of BrdU at E16 or E18, and embryos were sacrificed at E17 or E19 respectively. In order to study progenitor radial migration in the fetal cortex, BrdU injections to the dams were done 3 days before sacrifice (E17). Fetuses were thus harvested on E20, prior birth [26].

### Immunohistochemistry

**Figure 1:**
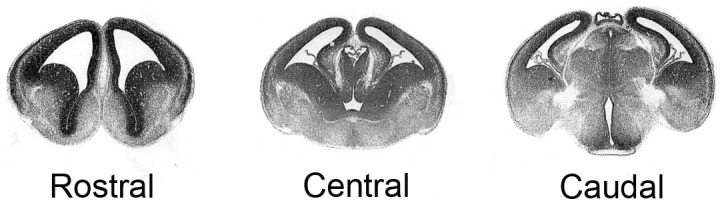
Representative images of the three cortical regions studied.

The fetal brains were removed and fixed for 1 h to 2H30 (depending on age) in 4% paraformaldehyde before being stored in sucrose 20%. Brains were included in gelatin (gelatin 7.5 g/100 ml, sucrose 20 g/100 ml, PBS 0.1 M) and 7 µm transversal sections were cut. Three cerebral cortical regions were collected: rostral, central and caudal cortex (Figure 1). Fixed section were permeabilized with DAKO target retrieval solution (DAKO North America, USA) for 10 min at 95°C and 10 min at room temperature (RT), and subsequently incubated with blocking buffer (donkey serum 10% in TBS-Triton 0,1%) for 1 hr at RT. The sections were incubated with primary antibody overnight at 4°C and then with secondary antibody for 1 h at RT. The primary antibobies were: BrdU (AbD Serotech, Dusseldorf, Germany) at 1∶500, Ki67 (BD pharmingen, San Diego, USA) at 1∶50, Satb2 (AbCAm, Cambridge, UK) at 1∶100, Nestin (Millipore, Billerica, MA, USA) at 1∶200, Sox5 (Aviva systems biology, San Diego, USA) at 1∶200, Tuj1 (Covance, New Jersey, USA) at 1∶200, Sox2 (Santa Cruz, Texas, USA) at 1∶200, NeuN (Chemicon, Temacula, USA) at 1∶500, Tbr2 (AbCam, Cambridge, UK) at 1∶500. For the secondary antibodies, donkey anti-rat rhodamine-conjugated antibody was used at 1∶500 for Brdu staining (Jackson ImmunoResearch laboratories, West Grove, PA, USA). For Ki67, nestin, Satb2 and NeuN staining, a donkey anti-mouse FITC antibody was used at 1∶500 (Jackson ImmunoResearch laboratories, West Grove, PA, USA) and for Sox5 and tuj1 stainings, a donkey anti-rabbit antibody was used at 1∶1000 (Jackson ImmunoResearch laboratories, West Grove, PA, USA). For Sox2 staining, a donkey anti-goat FITC antibody was used at 1∶500 (Jackson ImmunoResearch laboratories, West Grove, PA, USA). For Tbr2, an Alexa Fluor 488 donkey anti-rabbit antibody was use at 1∶500 (Invitrogen, Paisley, UK). Finally, nuclei were counterstained with DAPI (Hoechst 33342, invitrogen, Paisley, UK) 1/2000 for 10 min at RT.

### Tunel labeling

Cell death was quantified at E17 by Terminal transferase dUTP Nick End Labeling (TUNEL) (in situ cell death detection kit, TMR red, Roche, Vilvoorde, Belgium). Fixed sections were incubated in sodium citrate solution at 0.1M for 10 minute at RT and then in the “enzyme solution” diluted at 1/10 in the “label solution” for 1 h at 37°C.

### Cell count and statistical analysis

Digital photomicrographs of the regions of interest were obtained with an Olympus FSX100 microscope or Olympus Fluoview 1000 confocal microscope. For cell proliferation, S-phase, cell death and cell fate experiments, 3 cortical regions were studied (rostral, central and caudal) (Figure 1) and for each cortical region, the number of labeled cells was counted in the VZ/SVZ of three adjacent sections. Three to nine animals were studied per condition and experiment. Each fetus was coming from a different liter.

For the quantification of the cell cycle exit index, brain slices were stained with antibodies against Ki67 and BrdU 24 h after BrdU injection. In the 3 cortical regions, BrdU-positive and BrdU positive/Ki67 negative cells in the VZ/SVZ cells were counted in three adjacent sections from six independent embryos. The cell cycle exit index corresponds to the percentage of BrDU-positive cells that exited the cell cycle (BrdU-positive, Ki67 negative) to the total number of BrdU-positive cells [25].

For migration studies, different subregions of the cerebral cortex were identified based on cell density: VZ/SVZ, intermediate zone (IZ) and cortical plate (CP). For each experimental condition, the number of BrDU-positive cells in the VZ/SVZ, IZ and CP was counted in three adjacent slices of each cortex region (rostral, central and caudal) from six independent embryos. Similarly, the proportions of satb2 positive cells (a marker of upper layers neurons) and sox5 positive cells (deep layers marker) was quantified in the SVZ-VZ, IZ and CP in order to determine if altered migration affected layer formation.

For radial glia study, the general aspect of glia scaffold was analyzed and compared between cortex of control and A1254-exposed animals.

A statistical analysis was performed using unpaired student's T test between control and experimental condition. For cell cycle exit, two-way ANOVA was used to compare treatment and cortical region. For cell proliferation and S phase study, three-way ANOVA was used for multiple comparisons of age, cortical region and treatment (PrismR software). Significance was set at p<0.05.

## Results

### PCBs and thyroxine levels

The 5 measured PCB congeners (PCB101, PCB118, PCB153, PCB138, and PCB180) were undetectable in the serum obtained from control pregnant animals whereas concentrations between 34.5 and 262 µg/L were found in the exposed group (table 1). In the fetal brains, PCB concentrations were very significantly higher in fetuses on the 19th day of gestation (E19) exposed *in utero* to Aroclor 1254 compared to controls (p<0.05) (table 2). The total T4 and free T4 serum levels were significantly decreased in PCB exposed dams on the 20th of gestation (p<0.001) (table 3) as it has been shown in previous studies [11].

**Table 1:** PCB congener concentrations (µg/L) in serum sampled from pregnant rats (E19) exposed to 6 mg/kg/day A1254.

Group	PCB 101	PCB 118	PCB 153	PCB 138	PCB 180
A1254	34.5±3.6***	161.8±11.8***	165.8±12.1***	262±18.0***	41.2±3.1***
Control	≤0.5	≤0.2	≤0.2	≤0.2	≤0.2

Data are mean ± SEM.

***P<0,001, versus control group.

**Table 2:** PCB congener concentrations (ng/g wet weight) in fetal brains (E19) exposed to 6 mg/kg/day A1254.

Group	PCB 101	PCB 118	PCB 153	PCB 138	PCB 180
A1254	20.7±4.4	70.8±19.0*	111.7±24.6*	105.6±21.4**	16.6±4.2*
Control	7.9±0.8	1.3±0.4	0.3±0.2	0.9±0.5	0

Data are mean ± SEM.

*P<0.05.

**P<0.01, versus control group.

**Table 3:** Total T4 and free T4 concentrations in serum sampled from pregnant rat (E20) exposed to 6 mg/kg/day A1254.

A1254 exposition	Total T4 (ng/ml)	Free T4 (pg/ml)
Exposed dam serum	23.0±1.7***	2.7±0.2***
Control dam serum	35.7±2.0	14.7±0.5

Data are mean ± SEM.

***p<0.001, versus control group.

### Cell proliferation

**Figure 2:**
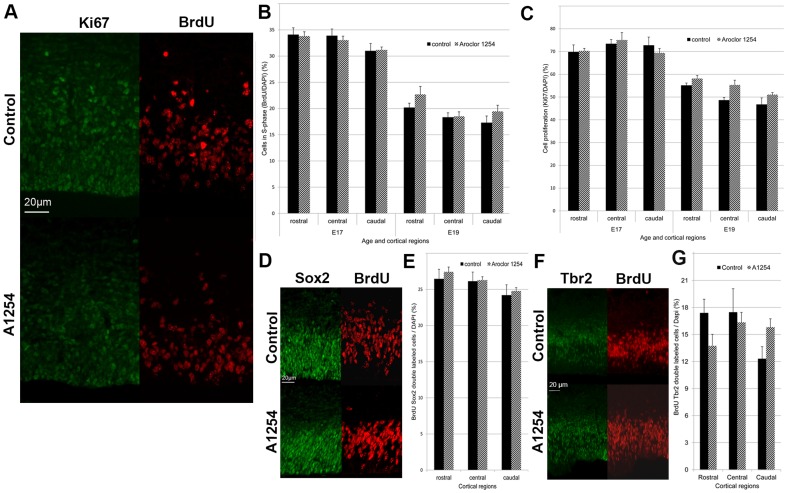
Aroclor 1254 does not affect neuronal progenitor proliferation. Evaluation of neuronal progenitor proliferation in the subventricular and ventricular zones of 3 cerebral cortical regions (rostral, central, caudal). (A) Representative images of confocal sections of SVZ and VZ of the caudal region of the cerebral cortex at E19 stained with Ki67 or BrdU 1 hour after injection in control and A1254 exposed animals. The ratio of BrdU-positive cells (B) and Ki67-positive cells (C) on all nuclei is calculated at E17 (n = 9 fetuses) and E19 (n = 3 fetuses). (D) Representative images of SVZ and VZ of the caudal region of the cerebral cortex at E17 stained with sox2 and BrdU 1 h after injection in control and A1254 exposed animals. (E) Ratio of BrdU-sox2 double labeled cells on all nuclei at E17 (n = 6 fetuses). (F) Representative images of SVZ and VZ of the caudal region of the cerebral cortex at E17 stained with Tbr2 and BrdU 1 h after injection in control and A1254 exposed animals. (G) Ratio of BrdU-Tbr2 double labeled cells on all nuclei at E17 (n = 6 fetuses). Data are mean ± SEM.

The effect of Aroclor 1254 on progenitor proliferation in the developing brain was studied using Ki67 and BrdU as markers of proliferation in the embryonic brain at E17 and E19. Ki67 is a cellular marker for proliferation labeling virtually any proliferating cell. The relative number of Ki67-positive cells in the SVZ-VZ (the ratio of Ki67 labeled cells on all cells labeled by DAPI), was unchanged after exposure to Aroclor 1254 at E17 and E19 in any region of the rostral, medial and caudal cortex. As expected, cell proliferation decreased with age (p<0.001) (Figure 2). BrdU labels cells in S-phase of the cell cycle at the time of injection. S-phase (synthesis phase) is the part of the cell cycle in which DNA is. One hour after BrdU injection, the relative number of BrdU-positive cells (the ratio of BrdU labeled cells on all cells labeled by DAPI) in the SVZ-VZ was unchanged at E17 and E19 in the Aroclor 1254 exposed animals, indicating no effect of PCBs on the proportion of cells in S phase (Figure 2). In order to determine whether a subpopulation of progenitors could be affected by prenatal exposure to A1254, we analyzed the proliferation of apical progenitors labeled by sox2 [27] and basal progenitors labeled by Tbr2 [28]. The ratio of BrdU-sox2 or BrdU-Tbr2 double-labeled cells on all nuclei was quantified at E17 1 h after BrdU injection to the dams (Figure 2). Proliferation of apical progenitors was not affected by prenatal exposure to A1254. Proliferation of basal progenitors was not significantly affected by exposure to A1254. However, exposure to A1254 caused a 28.5% increase in the number of basal progenitors in the caudal region that was close to significance (p = 0.058).

### Progenitor cell cycle exit

**Figure 3:**
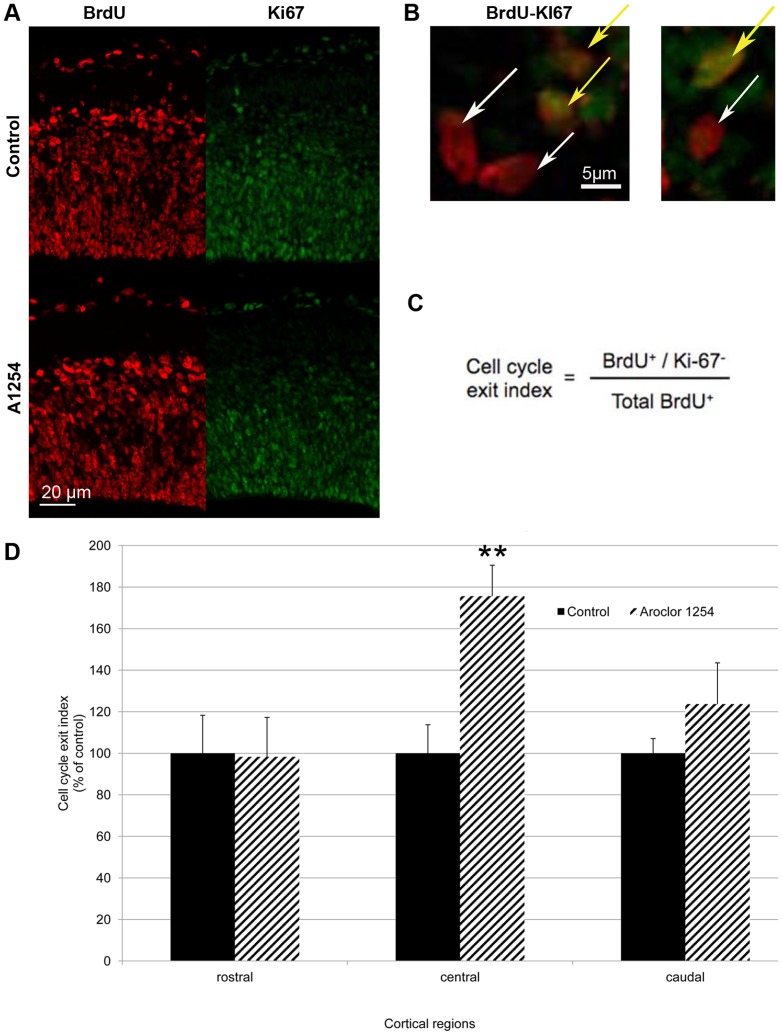
Aroclor 1254 increases cell cycle exit. Evaluation of cell cycle exit after fetal exposure to corn oil or Aroclor 1254. (A) Representative images of confocal sections of SVZ and VZ of the central region of the cerebral cortex at E17 stained with Ki67 and BrdU 24 hours after injection in control and A1254 exposed animals. (B) Higher magnification of the same representative images of confocal sections of SVZ and VZ of the central region of the cerebral cortex at E17 co-stained with Ki67 and BrdU 24 hours after injection. Cells that exited the cell cycle are BrdU positive and Ki67 negative (white arrow), whereas cells that are still in the cell cycle exit are positive for BrDU and Ki67 (yellow arrow). (C) Ratio used to calculate the cell cycle exit. (D) Quantitative analysis of the cell cycle exit ratio in SVZ and VZ at E17 in 3 cerebral cortical regions (rostral, central, caudal). Data are mean ± SEM (n = 6 fetuses for each group and region). **p<0.01, versus controls.

As described in the introduction some of the progenitors will leave the cell cycle after dividing and will start to migrate and differentiate. Since progenitor proliferation itself was not affected by exposure to PCBs, we aimed at evaluating progenitor cell cycle exit. The ratio of BrdU-positive and Ki67-negative cells on the total number of BrdU-positive cells allowed a relative quantification of cortical progenitors having left the cell cycle 24 h after being marked by BrdU (on E16 or E18) since they were Ki67 negative [25]. In comparison with the control group, the animals exposed *in utero* to Aroclor 1254 showed overall a 32% higher proportion of progenitors that had exited the cell cycle at E17 (p = 0.037). This effect appeared to be absent rostrally and maximal centrally, with an increase of 75% (p = 0.0039) (Figure 3). Time was also critical since at E19, the same quantitative analysis did no longer show any effect of exposure to Aroclor 1254 on cell cycle exit ratio (A1254 vs control, rostral: 21.6±4.0% vs 24.1±3.6%, central: 19.9±3.2 vs 25.6±4.7%, caudal: 24.5±4.9 vs 23.5±3.3%, mean±SEM).

### Neuronal Differentiation rate

**Figure 4:**
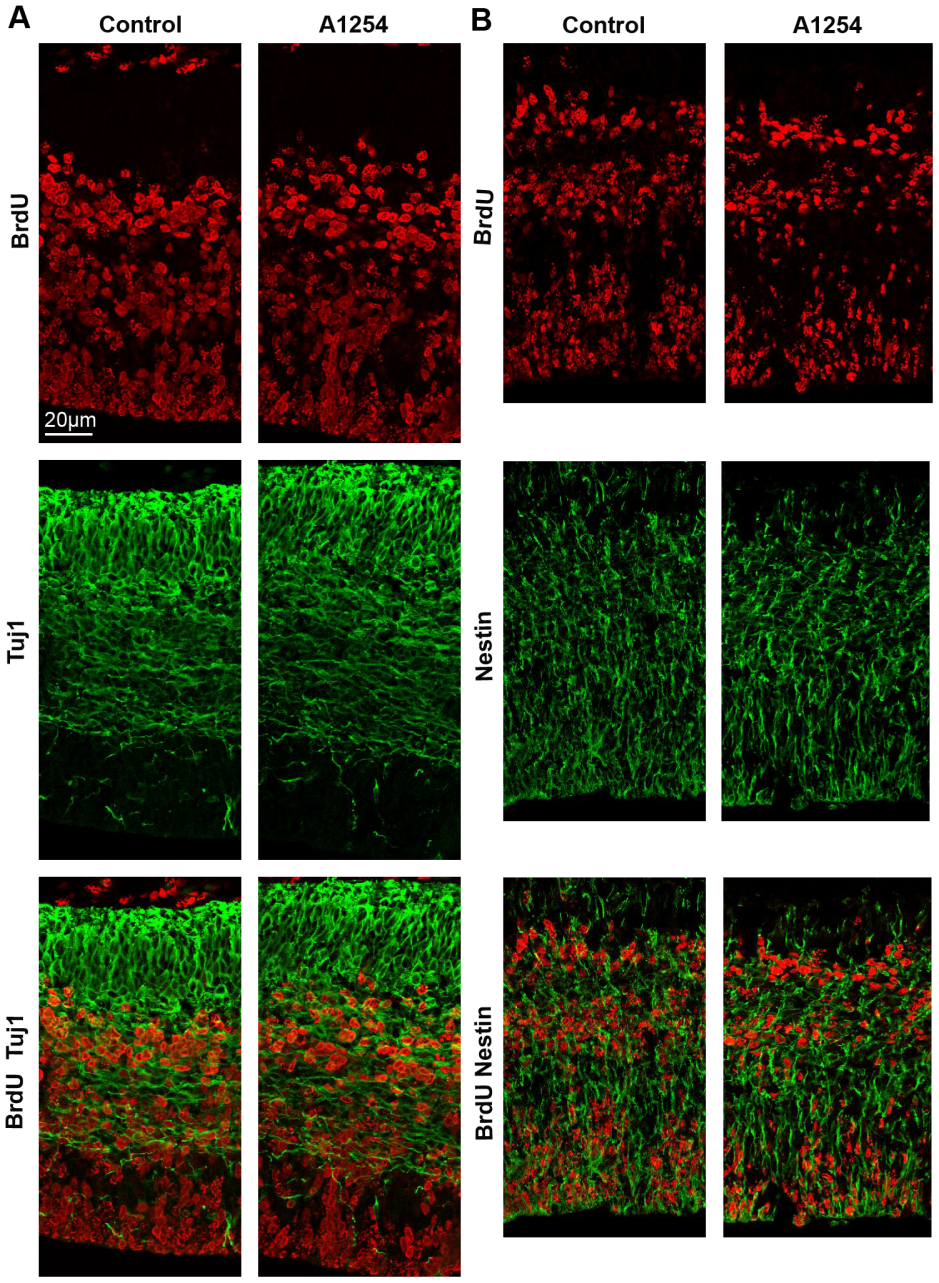
Aroclor 1254 does not affect neuronal differentiation rate. Evaluation of progenitor differentiation in the SVZ-VZ. (A) Representative images of confocal sections of SVZ and VZ of the central region of the cerebral cortex at E17 costained with Tuj1 (green) and BrdU (red) 24 hours after BrDU injection in control and A1254 exposed animals. (B) Representative images of confocal sections of SVZ and VZ of the central region of the cerebral cortex at E17 co-stained with Nestin (green) and BrdU (red) 24 hours after BrDU injection in control and A1254 exposed animals.

Since developmental exposure to Aroclor 1254 affected cell cycle exit, we analyzed the impact of PCBs exposure on progenitor differentiation by using nestin and tuj1 labeling at E17 (Figure 4). Nestin is specifically expressed by neuronal precursor cells and was used to quantify the proportion of BrdU labeled cells that were still dividing in the cerebral cortex, 24 hours after BrdU injection. Tuj1 or Neuron-specific class III beta-tubulin is a constituent of microtubules that is specifically expressed by mature neurons. Tuj1 was used to quantify the proportion of BrdU labeled cells that were already mature 24 hours after BrdU injection. The proportion of BrdU positive cells that were still proliferating (73±2% vs 75±1%, A1254 vs control, mean±SEM) and those that were already differentiating into neurons (1±0.1% vs 2±0.3%, A1254 vs control, mean±SEM) was similar in the control and exposed groups, indicating that, at that stage, cell differentiation rate was not affected by prenatal exposure to Aroclor 1254 (n = 6).

### Cell death

**Figure 5:**
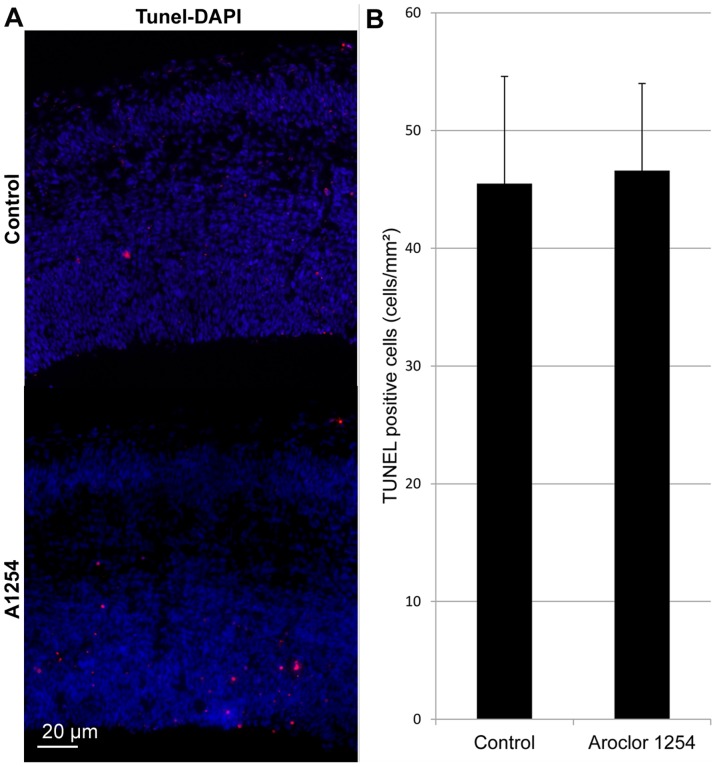
Aroclor 1254 does not affect cell death. Evaluation of cell death in the SVZ-VZ on E17. (A) Representative images of confocal sections of SVZ and VZ of the caudal region of the cerebral cortex at E17 stained with TUNEL (red) in a control and a A1254 exposed animal. (B) Average number of TUNEL-positive cells per mm^2^. Data are mean ± SEM (n = 6 fetuses for each age group).

Cell death was quantified at E17 by TUNEL labeling. The number of TUNEL positive cells was quantified in the VZ-SVZ (Figure 5). We did not observe any effect of exposure to Aroclor 1254 on cell death at E17 in the VZ–SVZ of the cerebral cortex (46.6±7.4 vs 45.5±9.1 cells/mm^2^, A1254 vs control, mean±SEM).

### Radial neuronal migration

**Figure 6:**
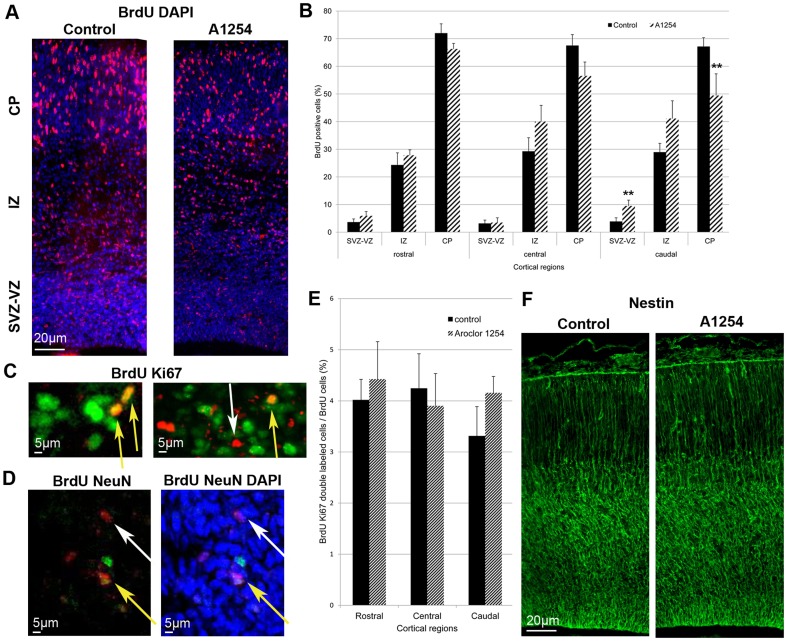
Aroclor 1254 delays radial neuronal migration. Quantification of radial neuronal migration after corn oil or Aroclor 1254 exposure. (A) Representative image of the subventricular-ventricular zone (SVZ/VZ), the intermediate zone (IZ) and the cortical plate (CP) of the caudal region of the cerebral cortex in a control and an A1254 exposed animal at E20. The section was co-stained with DAPI (blue) and BrdU (red) 3 days after injection. (B) Relative percentage of the BrdU-positive cells in the different layers (CP, IZ, SVZ-VZ) of the cerebral cortex at E20 in 3 cerebral cortical regions (rostral, central, caudal) (n = 6). (C) Representative image of a confocal section of the SVZ and VZ of the caudal region of the cerebral cortex of an A1254-exposed animal at E20 costained with Ki67 (green) and BrdU (red) 3 days after injection. Cells that still proliferate are positive for BrDU and Ki67 (yellow arrow). White arrows indicate BrDU-positive cells that do not express Ki67. (D) Representative image of a confocal section of the SVZ/VZ of the caudal region of the cerebral cortex in an A1254-exposed animal at E20. The section was co-stained with BrdU (red), NeuN (green) and DAPI (blue) 3 days after injection. BrDU-positive cells that remained in the VZ/SVZ after A1254 exposure and have differentiated express NeuN (yellow arrow). White arrows indicate BrDU-positive cells that do not express NeuN. (E) Ratio of BrdU-Ki67 double labeled cells on all Brdu positive cells at E20, 3 days after BrdU injections (n = 6). (F) Representative image of radial glia scaffold in a control and an A1254 exposed animal at E20. Data are mean ± SEM (n = 6 fetuses for each region). *p<0.05, versus controls.

**Figure 7:**
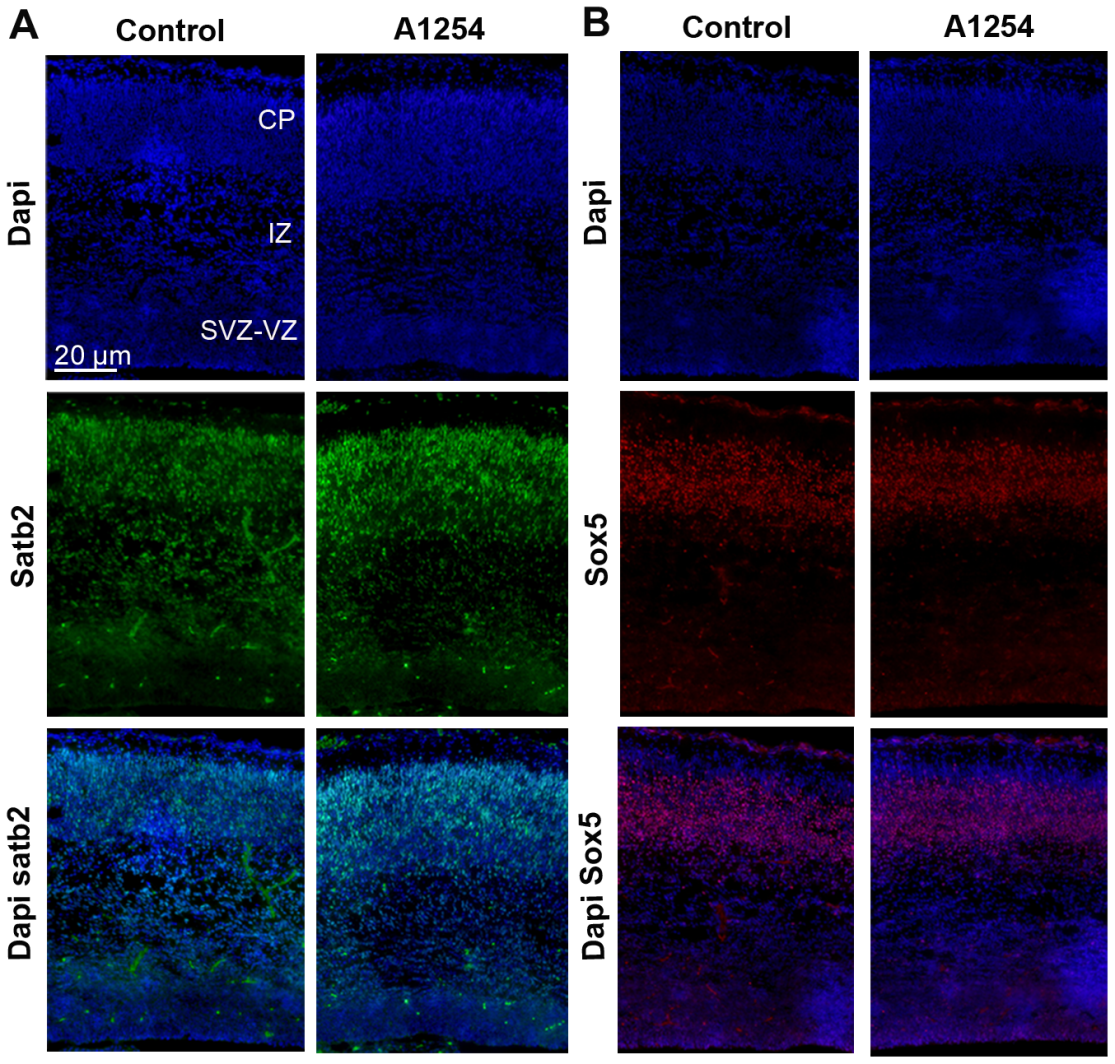
Aroclor 1254 does not affect cortical laminar organization. (A) Representative images of confocal sections of the caudal region of the cerebral cortex at E20 co-stained with dapi (blue) and Satb2 (green) in control and A1254 exposed animals. (B) Representative images of confocal sections of the caudal region of the cerebral cortex at E20 co-stained with dapi (blue) and Sox5 (red) in control and A1254 exposed animals.

During cortex development, neuronal progenitors migrate progressively from the VZ-SVZ toward the cortical plate (CP). In order to determine whether PCBs affect radial migration of projection neurons, we examined the position of BrdU-positive cells in the VZ-SVZ, the intermediate zone and the cortical plate, three days after BrdU injection. The allocation and number of BrdU-labeled cells in the different layers of the cerebral cortex of Aroclor 1254-exposed animals were analyzed and compared with the control brains. At E20, in the caudal cortex, the distribution of BrdU-positive cells showed a higher relative proportion of cells in the SVZ and VZ (p = 0.046) in exposed animals compared to the control animals whereas in the CP a reversed distribution was observed (p = 0.043). Thus, in exposed animals, fewer cells appeared to have reached the CP. These observations suggest a delay or a reduction of radial neuronal migration in this cortical region after developmental exposure to Aroclor 1254. In the two other cortical regions, we observed a similar though not significant trend towards a delayed radial neuronal migration after exposure to Aroclor 1254 (Figure 6). In order to clarify the nature of these BrdU positive cells retained in the VZ/SVZ, Ki67 and BrdU immunostainings were done at E20, 3 days after BrdU injections to the dams. Only 4% of the BrdU-positive cells were found to be Ki67 positive in the VZ/SVZ region (Figure 6) (n = 6), indicating that the vast majority of the delayed cells were not proliferating progenitors anymore. Similarly, NeuN and BrdU immunostainings were done at E20, 3 days after BrdU injections to the dams (Figure 6). Only 5% of the BrdU-positive cells were labeled with NeuN in the VZ/SVZ region, indicating that very few of the cells retained in the VZ/SVZ after exposure to Aroclor 1254 had differentiated into mature neurons (Figure 6) (n = 6).The laminar organization of the cortex was not affected at E20 since the proportions of satb2 positive cells (a marker of upper layers neurons) and sox5 positive cells (deep layers marker) in the SVZ-VZ, IZ and CP of the caudal cortex were not different between control and exposed animals (table 4 and 5; figure 7) (n = 6). Since prenatal exposure to Aroclor 1254 led to a delay of radial migration, we analyzed radial glia scaffold organization. General aspect of radial glia scaffold did not appear to be disrupted by exposure to PCB at E20 (figure 6).

**Table 4:** Relative percentage of Satb2 positive cells in the different layers of the caudal cortex at E20.

Condition	SVZ/VZ	IZ	CP
A1254	0.1±0.1 (1.7±1.7)	51.3±5.1 (445.5±65.9)	48.6±5.0 (440.8±106.6)
Control	0.1±0.1 (1.4±0.9)	41.9±3.0 (419.3±50.7)	58.0±3.0 (615.0±127.3)

Average number of cells per animal and region are expressed between brackets.

Data are mean ± SEM, versus control group.

**Table 5:** Relative percentage of Sox5 positive cells in the different layers of the caudal cortex at E20.

Condition	SVZ/VZ	IZ	CP
A1254	16.5±2.4 (201.7±35.2)	9.4±0.7 (111.7±8.1)	74.1±2.6 (877.8±27.5)
Control	13.6±3.2 (163.2±35.6)	9.4±0.9 (113.7±14.3)	76.9±3.2 (914.5±67.8)

Average number of cells per animal and region are expressed between brackets.

Data are mean ± SEM, versus control group.

## Discussion

Overall, this study provides the first evidence that fetal exposure to a PCB mixture causes alterations in cerebral cortical development that bear on particular aspects i.e. progenitor cell cycle exit and neuronal migration. Moreover such effects appear to be time- and region-specific. It is possible that PCB disruption of TH action plays a role in these observations.

As expected, the administration of Aroclor 1254 to dams during pregnancy results in a significant accumulation of PCBs in the serum of pregnant rat and in the brain of the offspring. These results confirm that PCBs easily cross the placental and the blood brain barrier. However, following a protocol of exposure that is commonly used in other studies [9], [29], PCB concentrations were higher than the rare values reported in humans exposed to these environmental pollutants in non-experimental conditions [3], [30], [31].

It is postulated that PCBs developmental neurotoxicity results partially from endocrine disruption. We show here that developmental exposure to Aroclor 1254 produces a significant decrease in serum total and free T4 in the exposed pregnant rats. This finding is consistent with previous studies showing a similar decrease in humans and animals serum during pregnancy [32]–[36]. Moreover, TH are essential for fetal brain development [37] and they are mainly supplied by the pregnant mother through the placenta during the first part of gestation [38].

We did not find any impact of developmental exposure to PCBs on progenitor cell proliferation in fetal brain. The lack of effects of prenatal exposure to PCBs on cell proliferation can be linked to the weak involvement of TH on cell proliferation in the cerebral cortex. Indeed, it has been shown that progenitors proliferation in the SVZ of the cortex is not affected by experimental hypothyroidism [39], [40] while it is modified in the cerebellum and hippocampus [39], [41]. Triiodothyronine (T3) regulates the expression of different genes coding for transcription factors, growth factors and cell surface receptors implicated in cell proliferation [17]. However, some studies showed that the activity of type 2 deiodinase is increased in rat fetal brain after exposure to PCBs which could explain that T3 brain level are maintained by increasing conversion of T4 catalyzed by deiodinase [35] based on the reduced serum thyroxine levels in pregnant dams, however, it is still possible that T3 levels are reduced locally.

Developmental exposure to PCBs increases cell cycle exit, a finding that had never been reported so far. Cell cycle exit is of critical importance for corticogenesis since it tightly controls the neuronal output throughout development. Nestin and Tuj1 immunolabelings did not suggest any effect of PCBs on cell differentiation. However, it is important to note that Tuj1 labels mature neurons. Thus, using this technique, we might have missed an effect of PCBs on intermediate stages of differentiation. Moreover, 24 hours after BrDU injection, very few BrDU-positive cells were already expressing Tuj1 (around 1%). An effect on differentiation might have been observed 48 hours after BrDU administration. Previous studies reported that T3 inhibits progenitor cells proliferation and induces differentiation into oligodendrocytes [42]. These observations are not consistent with our findings since we observed a decrease of thyroxine serum concentration associated with an increase of cell cycle exit. However, besides reducing serum thyroxine concentration, PCBs can directly interact with TH receptors [43]. Zoeller et al. reported that PCB metabolites could have TH agonist effects possibly involved in direct actions of PCBs in the fetus [11], [32], [33], [36]. In this context, PCBs effects on cell cycle exit could be explained by a direct TH agonist effect of PCBs metabolites.

Different studies showed direct toxicity of PCBs in vitro. PCB exposure increases apoptosis in neuronal cell culture through bcl2, bax and caspase 3 pathways [44]. We did not observe any increase in cell death in the SVZ-VZ after exposure to PCBs. Thus, the increase of cell cycle exit was not compensated by an increase of cell death which could have explained the absence of effect on cell differentiation.

We have shown that prenatal exposure to Aroclor 1254 alters the pattern of neuronal migration during cortical development. At E20, BrDU-labeled cells seem to accumulate in the SVZ-VZ of the caudal cortex in the Aroclor 1254-exposed group compared with the control group. Consistently, the number of BrdU-labeled cells that reached the cortical plate at E20 was lower in the Aroclor 1254 exposed animals. The cells that were retained in the VZ/SVZ after exposure to Aroclor were not mitotic anymore but did not appear to have completed differentiation into neurons since very few of them expressed Ki67 or NeuN, respectively. We suggest that PCBs might slow down radial neuronal migration. This delay might be explained by the importance of TH in the control of neuronal migration. Models of maternal hypothyroxinemia showed an alteration of tangential and radial neuronal migration [45]. Experiments with GFP-neurons showed that alterations of migration caused by hypothyroxinemia do not result from anomalies of the migratory neurons themselves but from undefined cues responsible for the guidance of their migration [45]. Actin polymerization depends on TH and is necessary to recognize laminin present in the extracellular matrix and induce neuronal migration [46]. T4 also regulates the production and extracellular deposition of laminin on the surface of astrocytes that influence neuronal migration in the developing brain [47]. Our results indicate that delayed migration was not due to alteration of the radial glia scaffold. However, the effects of developmental exposure to PCBs on neuronal migration are subtle and do not appear to affect the cortical layers defined by sox5 and satb2 expression. Cortical layers establishment is a process which lasts several days and continues during early postnatal life. Further work is required to understand the impact of PCBs on cortical layers formation at different ages during postnatal period.

This alteration of neuronal migration is only observed in the caudal region of the brain while the increase of cell cycle exit appears mainly in the central region. Other studies showed region-specific effects of PCBs. For example, the expression of HES1, a factor inhibiting neurogenesis and promoting gliogenesis, is increased by developmental exposure to PCBs but only in the lateral and rostral regions of the cortex [33]. Moreover, PCBs alter cell cycle exit in a critical age window since the increase of cell cycle exit is observed at E17 but not at E19. These age- and region-specific effects could be related to the developmental timing of these cortical regions. As cortical development follows a rostro-caudal progression, a region could be more sensitive than another at a specific time. Some PCB congeners can also bind arylhydrocarbon receptor (AhR) and this receptor seems to play a role in PCB toxicity [9], [48]. Region-specific effects of PCB could be explained by the AhR cortical distribution. Further studies could evaluate the later impact of perinatal exposure to PCBs on cerebral development by studying synaptogenesis and neurogenesis in the SVZ.

Neurogenesis and circuit development are regulated by thyroid hormones, which can have life-long impacts on neuronal proliferation and survival [49]. Because neurogenesis is ongoing during early development as well as adulthood in the VZ/SVZ, this relatively homogenous cell population provides an insight into factors that can control or disrupt neurogenesis at various points in time. Exposure to PCBs early in life could have life-long consequences on brain function by altering the developmental connectivity of the brain. In addition to the early effects that we have reported here, developmental exposure to PCBs could have late (likely cell autonomous) effects on new neurons exposed early during development, or new neurons born months after EDCs exposure could be affected. The latter effect would likely be a non cell-autonomous effect, if toxicant levels are no longer detectable in serum or brain.

T3 is known to promote differentiation of oligodendrocyte progenitors as well as myelin synthesis [50]. In addition, hypothyroidism caused by gestational and lactational administration of propylthiouracil has been shown to decrease expression of myelin-related genes in the cortex postnatally [51]. One could hypothesize that hypothyroidism caused by exposure to PCBs could lead to altered oligodendrocyte differentiation. However, the promoting effect of T3 on oligodendrocytes seems to peak during the first and second week of postnatal life which was beyond the scope of our study on prenatal exposure to PCBs. Interestingly, Sharlin et al. have shown that perinatal exposure to Arcolor 1254 significantly reduced density of myelin-associated glycoprotein-positive oligodendrocytes in the corpus calosum and the anterior commissure but did not mimic all of the effects of hypothyroidism on white matter composition [10].

In conclusion, we have shown that developmental exposure to PCBs results in subtle abnormalities of critical processes of cerebral cortex development. We provide the first evidence that cell cycle exit is increased and neuronal progenitor migration is delayed after prenatal exposure to PCBs. Although these effects may be caused by disruption of TH action, they are unlikely resulting from simple inhibition or activation of TH receptors. This “mosaic” effect on TH action is consistent with previous observations [52]. Although PCB production has been banned for nearly 40 years, the human population remains exposed to these chemicals. Even a subtle impact on human health, distributed across 40 years, represents a very large public health problem. In order to construct a public health protective program to identify and eliminate such endocrine disruptors, we need to understand their actions. Given the complexity of PCB effects on brain development, it is likely that similar chemicals, such as polybrominated diphenyl ethers, also produce such complex effects on TH action.
